# Mesenchymal stem cells alleviate dexamethasone-induced muscle atrophy in mice and the involvement of ERK1/2 signalling pathway

**DOI:** 10.1186/s13287-023-03418-0

**Published:** 2023-08-04

**Authors:** Belle Yu-Hsuan Wang, Allen Wei-Ting Hsiao, Hoi Ting Shiu, Nicodemus Wong, Amanda Yu-Fan Wang, Chien-Wei Lee, Oscar Kuang-Sheng Lee, Wayne Yuk-Wai Lee

**Affiliations:** 1 Center for Neuromusculoskeletal Restorative Medicine, CUHK InnoHK Centres, Hong Kong Science Park, Hong Kong; 2 Musculoskeletal Research Laboratory, SH Ho Scoliosis Research Laboratory, Department of Orthopaedics and Traumatology, Faculty of Medicine, The Chinese University of Hong Kong, Shatin, Hong Kong; 3 Center for Translational Genomics and Regenerative Medicine Research, China Medical University Hospital, China Medical University, Taichung, 404327 Taiwan; 4 Department of Biomedical Engineering, China Medical University, Taichung, 404327 Taiwan; 5 Institute of Clinical Medicine, National Yang Ming Chiao Tung University, Taipei, Taiwan; 6 Department of Orthopedics, China Medical University Hospital, Taichung, 404327 Taiwan; 7 Li Ka Shing Institute of Health Sciences, The Chinese University of Hong Kong, Prince of Wales Hospital, Shatin, Hong Kong; 8 Joint Scoliosis Research Centre of the Chinese University of Hong Kong and Nanjing University, The Chinese University of Hong Kong, Shatin, Hong Kong; 9 Key Laboratory for Regenerative Medicine, Ministry of Education, School of Biomedical Sciences, Faculty of Medicine, The Chinese University of Hong Kong, Shatin, Hong Kong

**Keywords:** Mesenchymal stem cell, Muscle atrophy, Dexamethasone, Cell therapy

## Abstract

**Background:**

High dosage of dexamethasone (Dex) is an effective treatment for multiple diseases; however, it is often associated with severe side effects including muscle atrophy, resulting in higher risk of falls and poorer life quality of patients. Cell therapy with mesenchymal stem cells (MSCs) holds promise for regenerative medicine. In this study, we aimed to investigate the therapeutic efficacy of systemic administration of adipose-derived mesenchymal stem cells (ADSCs) in mitigating the loss of muscle mass and strength in mouse model of DEX-induced muscle atrophy.

**Methods:**

3-month-old female C57BL/6 mice were treated with Dex (20 mg/kg body weight, i.p.) for 10 days to induce muscle atrophy, then subjected to intravenous injection of a single dose of ADSCs ($1\times {10}^{6}$ cells/kg body weight) or vehicle control. The mice were killed 7 days after ADSCs treatment. Body compositions were measured by animal DXA, gastrocnemius muscle was isolated for ex vivo muscle functional test, histological assessment and Western blot, while tibialis anterior muscles were isolated for RNA-sequencing and qPCR. For in vitro study, C2C12 myoblast cells were cultured under myogenic differentiation medium for 5 days following 100 $\mu$M Dex treatment with or without ADSC-conditioned medium for another 4 days. Samples were collected for qPCR analysis and Western blot analysis. Myotube morphology was measured by myosin heavy chain immunofluorescence staining.

**Results:**

ADSC treatment significantly increased body lean mass (10–20%), muscle wet weight (15–30%) and cross-sectional area (CSA) (~ 33%) in DEX-induced muscle atrophy mice model and down-regulated muscle atrophy-associated genes expression (45–65%). Hindlimb grip strength (~ 37%) and forelimb ex vivo muscle contraction property were significantly improved (~ 57%) in the treatment group. Significant increase in type I fibres (~ 77%) was found after ADSC injection. RNA-sequencing results suggested that ERK1/2 signalling pathway might be playing important role underlying the beneficial effect of ADSC treatment, which was confirmed by ERK1/2 inhibitor both in vitro and in vivo.

**Conclusions:**

ADSCs restore the pathogenesis of Dex-induced muscle atrophy with an increased number of type I fibres, stronger muscle strength, faster recovery rate and more anti-fatigue ability via ERK1/2 signalling pathway. The inhibition of muscle atrophy-associated genes by ADSCs offered this treatment as an intervention option for muscle-associated diseases. Taken together, our findings suggested that adipose-derived mesenchymal stem cell therapy could be a new treatment option for patient with Dex-induced muscle atrophy.

**Supplementary Information:**

The online version contains supplementary material available at 10.1186/s13287-023-03418-0.

## Background

Glucocorticoid and synthetic analogs such as dexamethasone (Dex) are potent anti-inflammatory and immunosuppressive drugs. The catabolic effects of glucocorticoid as endocrine hormones released in response to stress conditions or prolonged use at high dose have been reported to be associated with skeletal muscle atrophy [1, 2]. For instance, patients with Cushing’s syndrome exhibit peripheral muscle weakness [3]. In the past few decades, the number of long-term oral glucocorticoid prescription has increased by over 30%[4]. Patients with congenital adrenal hyperplasia and Addison's disease might need to take long-term Dex treatment [5–7]. The resultant loss of skeletal muscle mass and muscle weakness might lead to impaired quality of life, increased risk of falls, decrease in wound healing, compromised lung function and so on.

Healthy skeletal muscle consists of two types of muscle fibres, slow-twitch type I fibres and fast-twitch type II fibres. Type I fibres, with low ATPase activities, which have higher oxidative capacity and relatively more resistant to fatigue [8]. Type II fibres can be further divided into type IIA, type IIB and IIX fibres. Type IIA fibres, with high ATPase activities, have both higher oxidative and glycolytic capacity. Type IIB and type IIX both have high glycolytic but low oxidative capacity. The muscle atrophy induced by glucocorticoid is generally characterised by reduction in the size of type II fibres [9] and muscle force [10]. Several signalling pathways have been proposed to explain the decreased rate of protein synthesis and increased rate of protein breakdown underlying muscle atrophy [11, 12]. Muscle RING finger 1 (MuRF-1) and muscle atrophy F-box (Atrogin-1/MAFbx) are two muscle-specific E3 ubiquitin ligases playing important roles in muscle atrophy, which can be activated by glucocorticoid [13]. As the underlying mechanisms are not fully understood, effective treatment for alleviating glucocorticoid-induced muscle atrophy is limited.

The paracrine and immunomodulatory properties of mesenchymal stem cells (MSCs) make it one of the popular cell types in cell therapy [14]. Over a thousand clinical trials using MSCs were designed to test therapeutic interventions for various severe diseases such as treatment in orthopedics, degenerative, immune rejection, inflammation and autoimmunity [15]. In addition to these known targeted medical conditions, recent study suggested that autologous transplantation of adipose-derived MSCs (ADSCs) with collagen hydrogel into crushed tibialis anterior muscle render beneficial effects in improving muscle function and regeneration [16]. Moreover, systemic injection of human ADSCs in animal model of muscle injury induced expression of human dystrophin without immunosuppression [17, 18]. With these, we hypothesised that that systemic injection of ADSCs could improve muscle mass and muscle functions in animal model of Dex-induced muscle atrophy. The signalling pathway involved in ADSC-mediated benefit would be explored to elucidate the underlying mechanisms.

## Methods

### Dex-induced muscle atrophy animal model

Three-month-old female C57BL/6 mice were used to develop the muscle atrophy model. To induce muscle atrophy, mice received daily intraperitoneal injection of Dex (20 mg/ kg body weight, Sigma #D4902) [19] for 10 days. Animals were housed in 12 h light/ 12 h dark cycles at 22–28 ℃ with standard chow diet and water. Five mice were kept in one standard small cage.

### In vivo treatments, ADSC transplantation and ERK1/2 inhibitor injection

Human adipose-derived mesenchymal stems (ADSC) were cultured following our previous publication using Iscove's modified Dulbecco's medium (IMDM) (Gibco #12200036) containing 10% foetal bovine serum (FBS) (Gibco #10270106) and 1% penicillin–streptomycin-glutamine (PSG) (Gibco #10378016) 10 ng/mL FGF2 (PeproTech #100-18B) [20]. Mice were randomly divided into non-Dex-treated group (CON), dexamethasone-treated group (DEX) and ADSC treatment group (DEX + ADSC). After 10 days of dexamethasone induction, mice received a dose of ADSCs ($1\times {10}^{6}$/kg body weight in 200 $\mathrm\mu$L PBS) or PBS through tail vein injection, respectively. Mice were killed with carbon dioxide asphyxiation 7 days after ADSC transplantation. For in vivo mechanistic study, mice received daily administration of MEK-ERK1/2 inhibitor U0126 (25 µM/kg body weight, i.p.) from day 11 for 7 days [21–24].

### C2C12 cell culture and treatments

C2C12 cells were cultured with high-glucose Dulbecco's modified Eagle medium (HG-DMEM) with 10% FBS and 1% PSG and followed with 5 days of differentiation (HG-DMEM medium with 2% horse serum (HS) (Gibco #16050122) and 1% PSG). Dex group (DEX) and ADSC treatment group (DEX + ADSC) received 4 days of 100 µM Dex (dissolved in DMSO and diluted with PBS) treatment with IMDM medium contained 10% FBS and 1% PSG or 100 µM Dex treatment with ADSC-conditioned medium. Control group (CON) was cultured with IMDM medium contained 10% FBS, 1% PSG and DMSO (equal volume as DEX and DEX + ADSC groups) for 4 days.

For ADSC-conditioned medium collection, $2\times {10}^{5}$ ADSCs were seeded in 6-well plate and cultured with IMDM medium containing 10% FBS, 1% PSG and 10 ng/mL FGF2. Two days after cell culture, conditioned medium was collected and centrifuged to remove cell debris. For inhibitor treatment, all inhibitors were dissolved in DMSO and freshly diluted with culture medium. 10 µM JNK inhibitor (SP-600125) [25, 26], 1 µM ERK1/2 inhibitor (PD-325901) [27, 28] or 10 µM p38 inhibitor (SB-203580) [29, 30] was added concurrently during ADSC-conditioned medium treatment. Meanwhile, equal volume of DMSO was added in DEX and DEX + ADSC groups.

### Body composition

Whole-body, hindlimb and forelimb lean mass and fat mass were measured by small animal dual-energy X-ray absorptiometry (DXA) (UltraFocus^DXA^, Faxitron Bioptics, Tucson, Arizona, USA) 7 days after ADSC transplantation. Dedicated Bioptics Vision software was used for data analysis.

### Muscle functional tests

Grip strength metre was used to measure the forelimb grip strength. Each mouse was allowed to grasp the machine, and then its tail was gently pulled back and the tension was recorded automatically by the machine. Each mouse had five times to perform the test, and 5-min rest was given between each test. The highest and lower results were excluded, and the remainder values were recorded and normalised with whole body weight for analysis. Rodent Treadmill (Ugo basile #47303) was used to measure the fatigue-like behaviour in mice. Training section and fatigue test were performed by following the published protocol [31]. Animals followed the 3-day-training protocol and one full day resting before starting the fatigue test. The test ended when the mouse remained in the fatigue zone for 5 times. Total running distance was recorded and present as the fatigue level. For the ex vivo muscle functional test, right hindlimb gastrocnemius (GA) muscles were carefully isolated from anaesthetised mice and tied at the Achilles’s tendon for mounting on Dynamic Muscle Control system (DMC v5.4; Aurora Scientific, Inc.) supplemented with Krebs buffer, 95% O_2_ and 5% CO_2_. The optimal length of the GA muscle was measured, a single 150 Hz stimulus was given for three times, and the responses were recorded for calculation of twitch force. For the determination of peak tetanic force, a continuous 150 Hz stimulus was given for three times and the responses were recorded. Normalised twitch force and peak tetanic force were achieved by dividing with GA muscle cross-sectional area (CSA). After resting for 5 min, the fatigability was measured by repeated stimulus every 5 s for a total of 300 s and the isometric contractions were recorded. A stimulus of 150 Hz was given to GA muscle 5 min and 10 min after repeated stimulus, and the peak tetanic forces were recorded to determine the recovery rate. The results were analysed using the Dynamic Muscle Analysis system (DMA v3.2; Aurora Scientific, Inc.).

### Histochemical and immunofluorescence staining

Freshly isolated GA muscle tissues from the left hindlimb were snap frozen in liquid nitrogen and kept at -80℃ until further processing. Frozen muscles were embedded in OCT embedding medium and sectioned at 10 µm for further staining. For immunofluorescence staining, samples were fixed in pre-ice methanol and blocked with 2% horse serum for 1 h at room temperature and followed by primary antibodies incubation overnight at 4℃ and secondary antibodies incubation at room temperature for 1 h. DAPI was used for nuclear staining. Hematoxylin and eosin (H&E) staining was used to obtain cross-sectional morphology of muscle fibre. ImageJ software was used for image data analysis. Fusion index was calculated as the number of nuclei presented inside each MyHC-positive myotube and divided by the total number of nuclei in a field of view.

### Western blot

RIPA buffer (Abcam #ab156034) with protease/phosphatase inhibitor (Thermo Scientific™ #1861821) was used to extract proteins from tissue and cell samples. Protein concentration was measured by using Pierce™ BCA Protein Assay Kit (Thermo Scientific™ #23225). Samples were separated in 10% SDS–polyacrylamide gel and transferred to PVDF membranes at 100 V for 1 h. Membranes were then blocked in 5% BSA at room temperature and incubated with primary antibodies at 4℃ overnight, followed by secondary antibodies incubation at room temperature for 1 h. Chemiluminescent substrate (Thermo ScientificTM #34095) was added, and membranes were placed in ChemiDoc MP imaging system (Bio-Rad) for signals detection. ImageJ software was used for image data analysis. Full blot images were provided in supplementary data (Additional file 1: Fig. S1).

### RNA extraction and qPCR

TRIzol (Invitrogen #15596018) was used for total RNA purification, and then High-Capacity cDNA Reverse Transcription Kit (Applied Biosystems™ #4368813) was used to obtain cDNA. Gene expression quantification was performed by using the QuantStudio™ 7 Flex Real-Time PCR System (Applied Biosystems™). All gene expression results were normalised by housekeeping gene GAPDH.

### Bulk RNA-sequencing

Total RNA was extracted from GA muscles as mentioned above. RNA was then lysed into short fragments using a frag- mentation buffer. First-strand cDNA was synthesised using random N6 primers, followed by second-strand cDNA synthesis. The ends of double cDNA were repaired; 5′ ends were phosphorylated, and 3′ ends formed cohesive ends with A-tailing. Then, cDNA was ligated to the sequencing adapters. The ligation products were amplified using BGISEQ platform to build a cDNA library and sequenced on the DNBSEQ PE100 platform. Raw data were preprocessed with quality control steps to remove the adapter signals during library preparation and low signal sequence quality reads. The quality control was performed with SOAPnuke (v 1.5.2). After quality control, the “clean data” were stored in FASTQ format for further analysis. Sequence alignment was performed with software HISAT2 (v2.0.4) and mapped to template GRCm38 (NCBI, GCF_000001635.26_GRCm38.p6). After the mapping procedure, the total mapping ratio and the uniquely mapping ratio of each sequenced data were examined to monitor the between sample quality control. Finally, the sequenced samples that passed the quality check were obtained for further analysis.

### Statistical analysis

Sample size for each experiment was calculated by G-power software. All statistical analyses were performed using SPSS software and statistical significance was determined using one-way ANOVA (*P < 0.05, **P < 0.01, ***P < 0.005, #*P* < 0.001). Each experiment was performed at least three times.

## Results

### ADSC treatment improve muscle mass and muscle atrophy-associated gene and protein expression

**Fig. 1:**
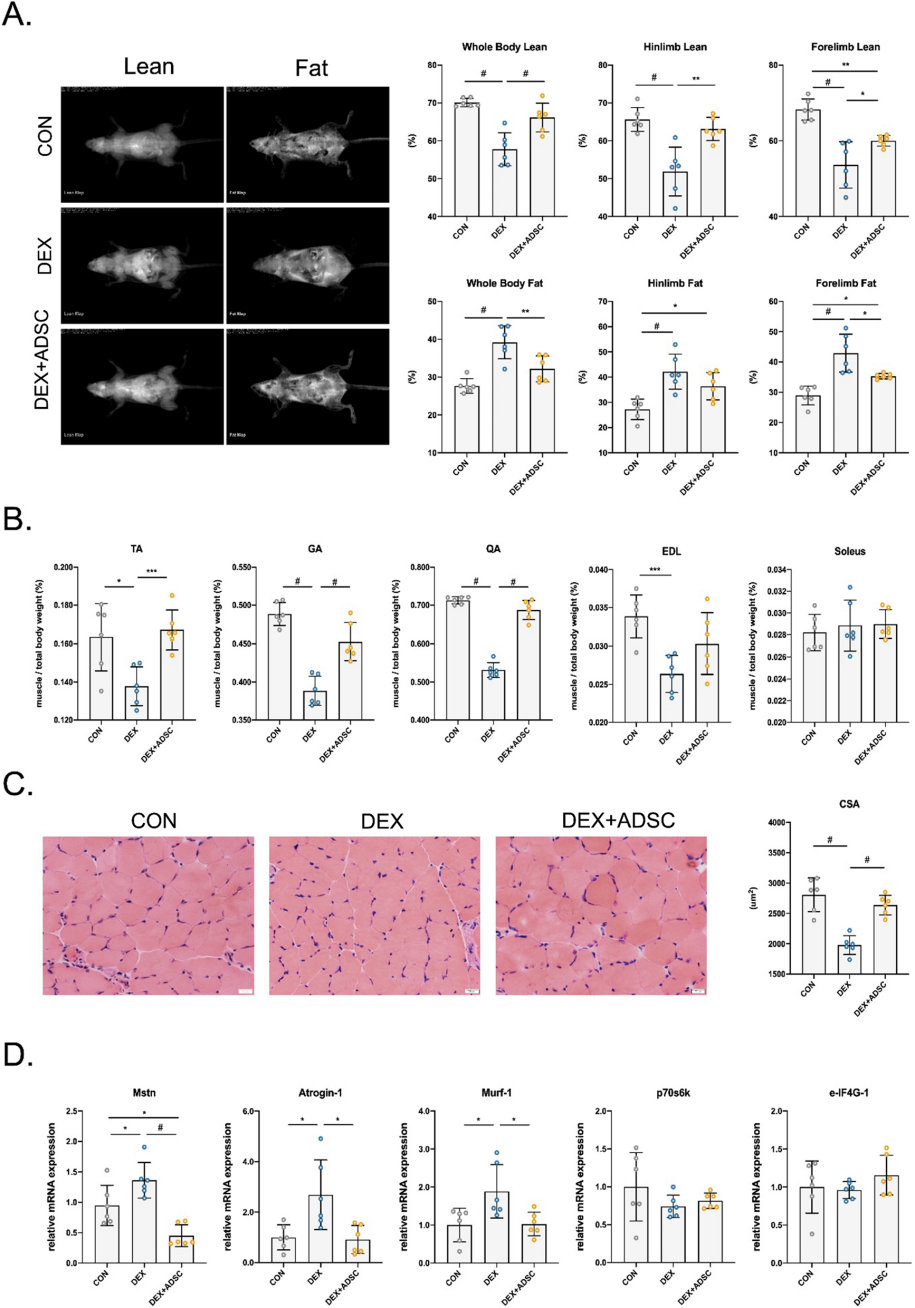
Assessment of muscle mass after ADSCs treatment in Dex-induced muscle atrophy mice. **A** Whole-body, hindlimb and forelimb composition including lean mass and fat mass measured by DXA machine. **B** Quantification of tibialis anterior (TA), gastrocnemius (GA), quadriceps femoris muscle (QA) extensor digitorum longus (EDL) and soleus muscle wet weight. **C** Quantification of muscle CSA in H&E stained GA muscle. **D** Analysis of muscle atrophy-related genes (Mstn, Atrogin-1 and Murf-1) and protein synthesis-related genes (p70s6k and e-IF4g-1) genes expression in TA muscles with ADSCs treatment. *n* = 6 per group. Quantitative data are presented as mean ± SD. Statistical analysis are performed using one-way ANOVA test, with significance set at *P* < 0.05 (*P < 0.05, **P < 0.01, ***P < 0.005, #*P* < 0.001)

The beneficial effects of ADSCs in skeletal muscle system were evaluated through multiple experiments. First, whole-body composition was measured by DXA machine. The results suggest that ADSC treatment reversed the loss of lean mass, including whole body, hindlimb and forelimb (Fig. 1A). Meanwhile, body fat which was increased by Dex can be alleviated by ADSC treatment. The wet weight of hindlimb muscle including tibialis anterior (TA), gastrocnemius (GA) quadriceps femoris (QA) and extensor digitorum longus (EDL) muscle was significantly increased by ADSC treatment (Fig. 1B). H&E staining results indicated that ADSCs are capable of reversing the GA muscle CSA (Fig. 1C). Dex is known to affect skeletal muscle through activating protein degradation and inhibiting protein synthesis. Thus, we performed the qPCR analysis for genes expressed in protein degradation and protein synthesis. Results showed that Dex activated genes involved in protein degradation (Mstn, Atrogin-1 and Murf-1) but had no effect on genes responsible for protein synthesis (p70s6k and e-IF4G-1) (Fig. 1D). To conclude, these results show that ADSCs have the ability in reversing the phenotypes in Dex-induced muscle atrophy.

### ADSC treatment improves muscle functions in Dex-induced mice

**Fig. 2:**
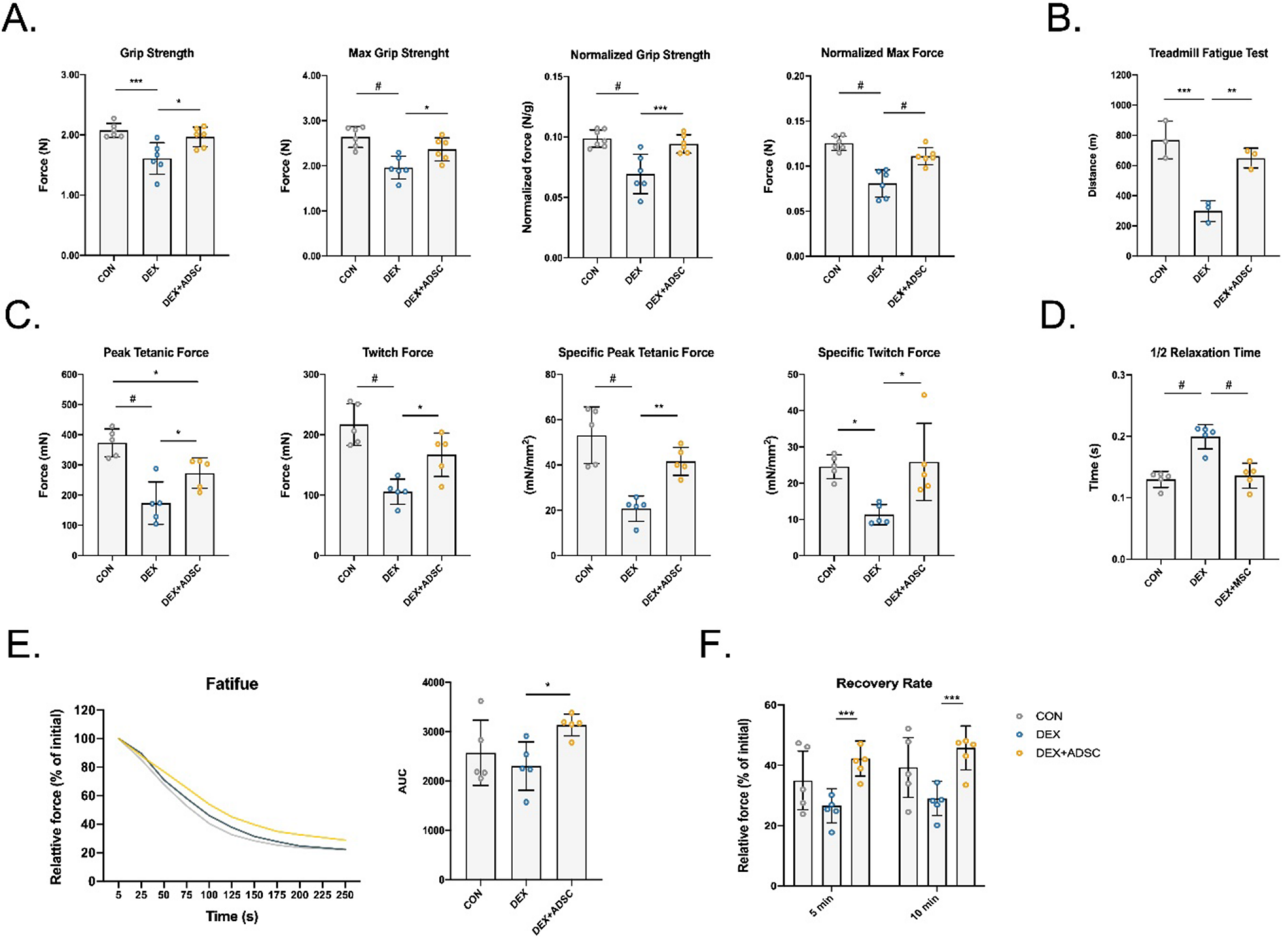
Effect of ADSCs treatment in improving skeletal muscle functions and regulating muscle fibre types. **A** Hindlimb grip strength were measured at 7 days after ADSCs treatment with or without normalised by whole body weight. *n* = 6 per group. **B** Treadmill fatigue were measured 6 days after ADSCs treatment. *n* = 3 per group. **C**–**F** Ex vivo GA muscle contraction test. *n* = 5 per group. Peak tetanic fore and twitch fore with or without normalised by GA muscle CSA **C** Half-relaxation time (**D**). GA muscle fatigability normalised by prefatigued developed tension (**E**). Contraction force of GA muscle after 5 min and 10 min of fatigue (**F**). *n* = 6 per group. Statistical analysis are performed using one-way ANOVA test, with significance set at *P* < 0.05 (*P < 0.05, **P < 0.01, ***P < 0.005, #*P* < 0.001)

**Fig. 3:**
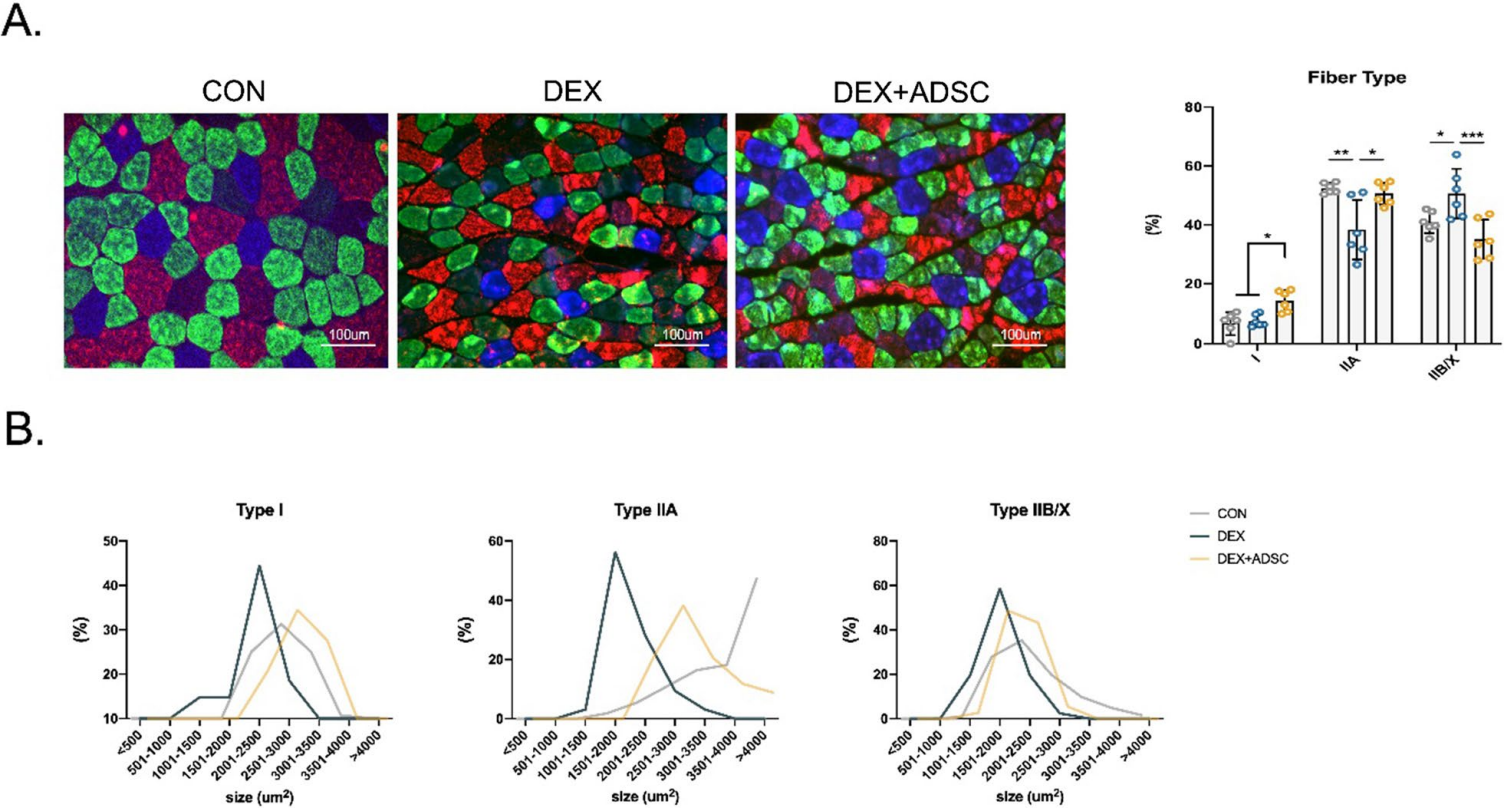
Effect of ADSCs treatment in controlling muscle fibre type switching (A-B) Immunofluorescence staining and quantification for fibre types in GA muscle sections. Blue: type I; green: type IIA; red: type IIB; black: type IIX staining (**A**). Fibre types CSA distribution (**B**). *n* = 6 per group. Statistical analysis are performed using one-way ANOVA test, with significance set at *P* < 0.05 (*P < 0.05, **P < 0.01, ***P < 0.005, #*P* < 0.001)

To further understand the beneficial effect of ADSCs on skeletal muscle functions, forelimb grip strength and ex vivo muscle functional test were performed. Forelimb grip strength and max grip strength were markedly increased in ADSC treatment group as compared with Dex-treated mice; similar results were also shown in the normalised grip strength (Fig. 2A). Walking distance is one of the muscle function parameters; our treadmill running fatigue test result suggested that total running distance can be significantly improved after ADSC treatment (Fig. 2B). *Ex vivo* muscle functional test showed that ADSC treatment group has a better force response to electrical stimulations (Fig. 2C). Compared with Dex-induced mice, ADSC-treated mice produced ~ 57% more peak tetanic force and ~ 58% more twitch force in GA muscle. The half-relaxation time (time taken for force to decline from 50 to 25% of the peak force) was found ~ 35% longer in Dex-induced mice compared with control group, but ADSC treatment group had ~ 31% shorter half-relaxation time (Fig. 2D). After receiving continuous stimulation, no difference between control mice and Dex-induced mice had found (Fig. 2E). However, ADSC-treated mice showed significantly improved anti-fatigue ability. Also, better recovery rates were observed in ADSC-treated mice (Fig. 2F). Taken together, these data suggest that ADSCs reverse the loss of muscle functions caused by Dex and enhance the resistance to fatigue ability. Skeletal muscle fibre type has been known to contribute to muscle contraction. Therefore, we next checked the fibre type population in each group. The number of oxidative type I muscle fibre significantly increased after ADSC treatment; meanwhile, ADSCs also reversed the change of type IIA and type IIB/X fibres (Fig. 3A). The distribution of CSA of type I muscle fibre also shifted towards a larger size compared to DEX group (Fig. 3B). As previous studies have mentioned, oxidative type I muscle fibres are more fatigue resistant than type II muscle fibres. Thus, ADSC increased type I muscle fibre number and CSA explain the enhancement in GA muscle anti-fatigue ability.

### Signalling pathways regulated by ADSCs in Dex-induced mice

**Fig. 4:**
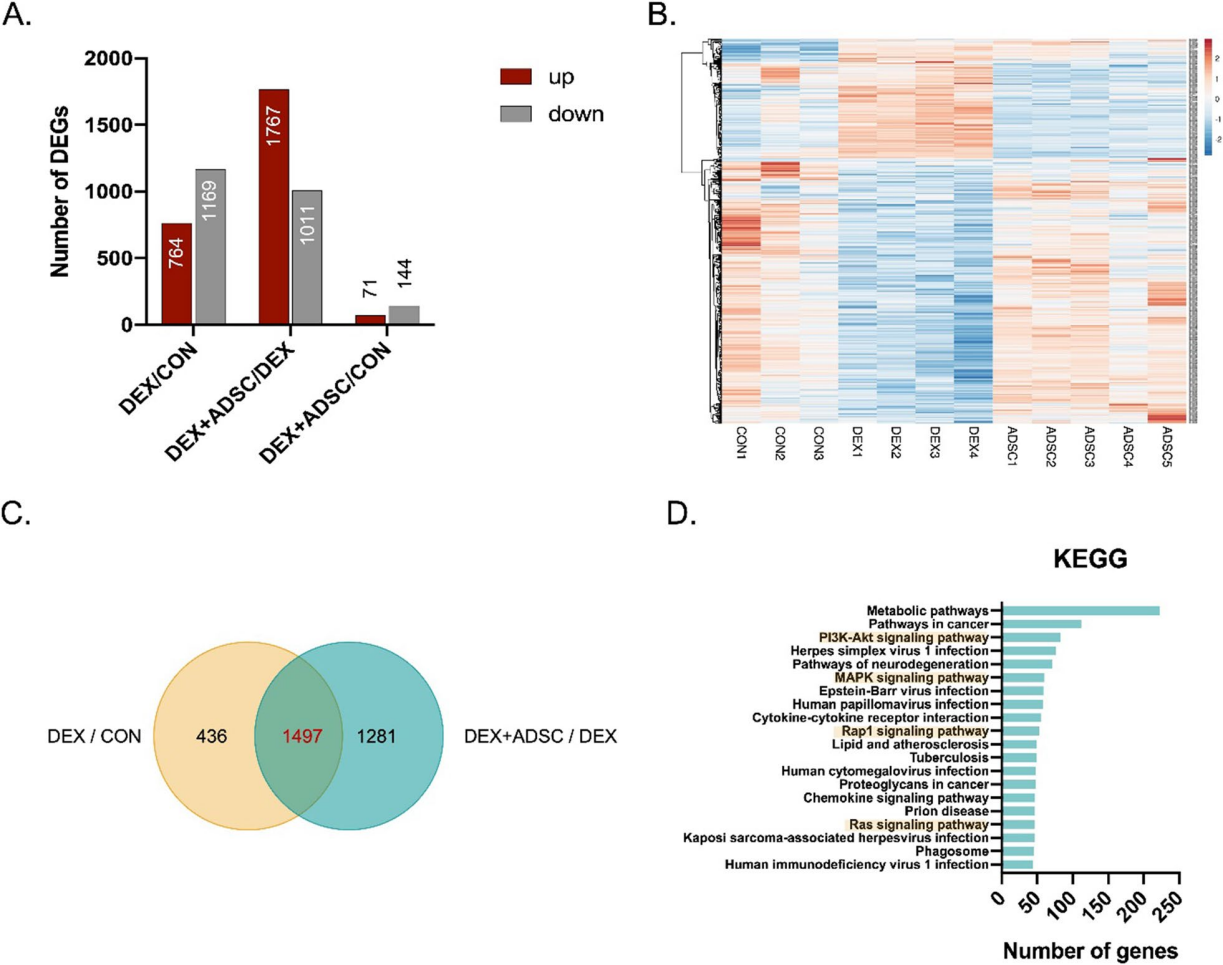
RNA sequencing of GA muscle from control, Dex-induced muscle atrophy mice and ADSCs-treated mice. **A** Differentially expressed genes (DEGs) between DEX vs CON, DEX + ADSC vs DEX and DEX + ADSC vs CON. **B** Heatmap of DEGs in different samples. **C** Venn diagram of the overlapped DEGs between DEX vs CON and DEX + ADSC. **D** Top 20 functionally enriched KEGG pathway analysis of differentially expressed genes (DEGs) found in DEX + ADSC vs DEX

To determine the roles of ADSCs in improving muscle mass and muscle functions, we performed the RNA-Sequencing transcriptomic analysis. There were 1933, 2778 and 215 differentially expressed genes (DEGs) found in DEX vs CON, DEX + ADSC vs DEX and DEX + ADSC vs CON, respectively (Fig. 4A). The results suggested that DEX + ADSC group is more similar to the CON group, which has much lower DEGs compared with DEX group (Fig. 4B). There are 77.4% of the DEGs regulated by DEX could also affected by ADSCs (1497 out of the 1933 DEGs) (Fig. 4C). Next, we focused on the 2778 DEGs that were regulated by ADSCs. For the purpose of acquiring the functional classification of these 2778 DEGs, KEGG pathway enrichment analysis was performed (Fig. 4D). The results showed that PI3K-Akt, mitogen-activated protein kinases (MAPK), Rap1 and Ras signalling pathway were found to be listed in the top 20 functional enriched KEGG pathways. In skeletal muscle, MAPK was known as one of the major regulators in response to oxidative, energetic and mechanical stress [32]. Thus, we further investigated the role of MAPK in regulating skeletal muscle functions by ADSCs.

### Effects of MAPK signalling in Dex-treated C2C12 myotubes

**Fig. 5:**
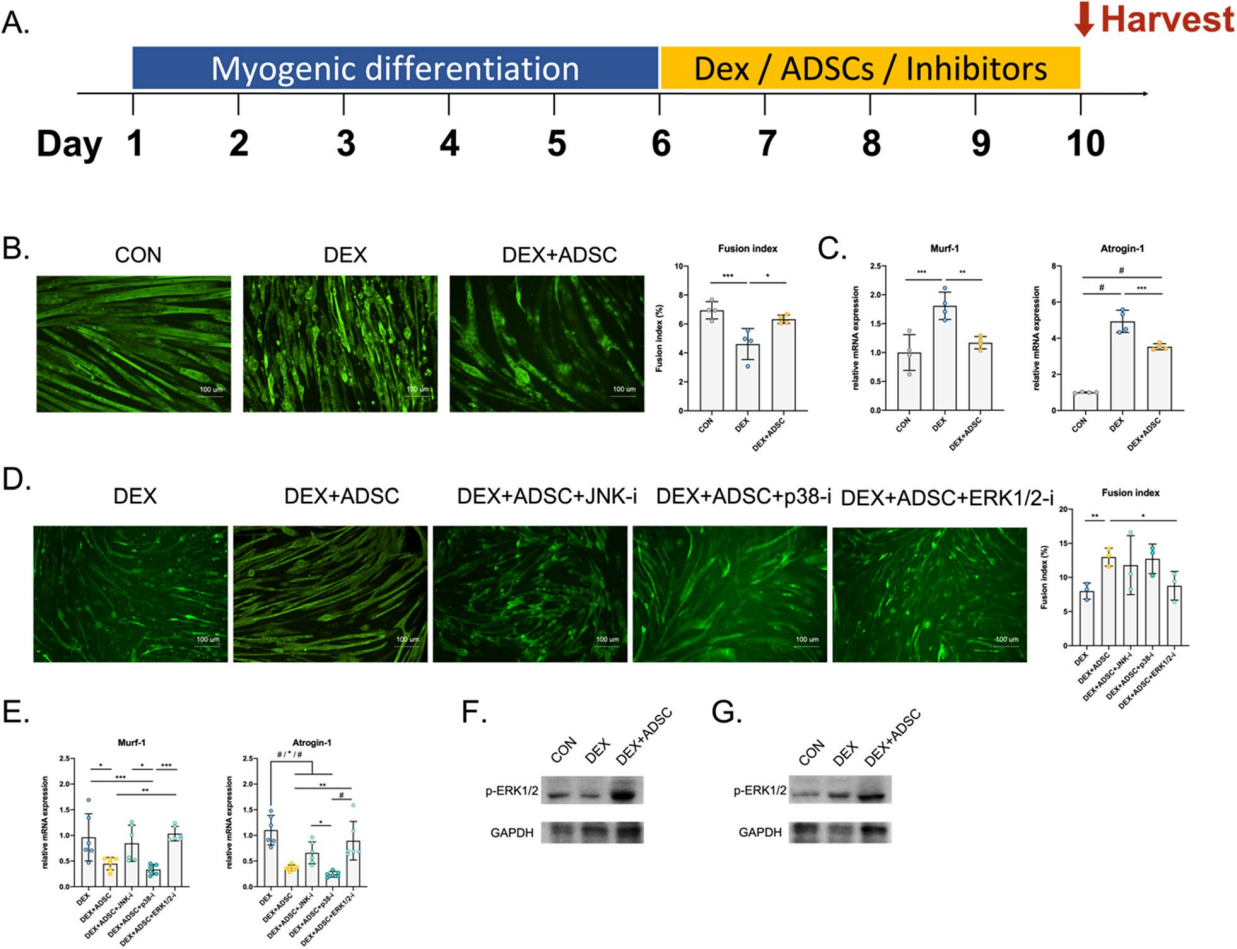
Inhibition of MAPK signalling pathway in Dex-induced muscle atrophy C2C12 cells. **A** A schematic diagram for in vitro cell experiment. (B-C) The establishment of Dex-induced C2C12 cells as a muscle atrophy in vitro model. n = 4 per group. **B** Immunofluorescence staining for myosin heavy chain and the quantification for myotube fusion index. **C** Real-time PCR analysis of muscle atrophy genes. **D** The myosin heavy chain staining and fusion index calculation of ADSCs-treated atrophy C2C12 cells with MAPK inhibitors (JNK, p38 and ERK1/2). *n* = 3 per group. **E** Real-time PCR analysis of muscle atrophy genes for samples treated with inhibitors. *n* = 6 per group. **F**, **G** ERK1/2 protein expression for in vitro (**F**) and in vivo (**G**) samples. Quantitative data are presented as mean ± SD. Statistical analysis are performed using one-way ANOVA test (A-B, D) and unpaired t-test (**C**), with significance set at *P* < 0.05 (*P < 0.05, **P < 0.01, ***P < 0.005, #*P* < 0.001)

To validate the RNA-seq results, we further used an in vitro model to study the necessity of MAPK for ADSC treatment. C2C12 myotube treated with high dosage of Dex was used as an in vitro muscle atrophy model [33, 34]. After treated with ADSC-conditioned medium for 4 days, myotube morphology and muscle atrophy gene expression was significantly reversed (Fig. 5A, B). To confirm the necessity of MAPK signalling pathway for ADSC treatment, Dex-induced C2C12 myotubes with ADSC-conditioned medium were cultured with JNK, p38 or ERK1/2 inhibitor. After inhibiting ERK1/2 expression, the effect of ADSCs on improving myotube morphology was suppressed but no significant effect after treated with JNK or p38 inhibitor (Fig. 5C). Furthermore, muscle atrophy genes, Murf-1 and Atrogin-1, were significantly upregulated after inhibition of ERK1/2 compared with ADSC-treated group and no significant differences were found after treated with JNK or p38 inhibitors (Fig. 5D). These in vitro results suggest that ADSCs might improve Dex-induced muscle atrophy through ERK1/2 pathway. Next, we measured the ERK1/2 protein expression level in both muscle tissues and C2C12 myotube samples to confirm our hypothesis (Fig. 5E, F, Fig. S1). Western blot results showed that higher levels of phosphorylated ERK1/2 protein were found in ADSC treatment group in both in vivo and in vitro samples. Ultimately, ERK1/2 was confirmed as one important pathway for ADSC in revering Dex-induced muscle atrophy.

### Ablation of ERK1/2 in ADSC-treated Dex-induced mice leads to a decrease anti-fatigue ability

**Fig. 6:**
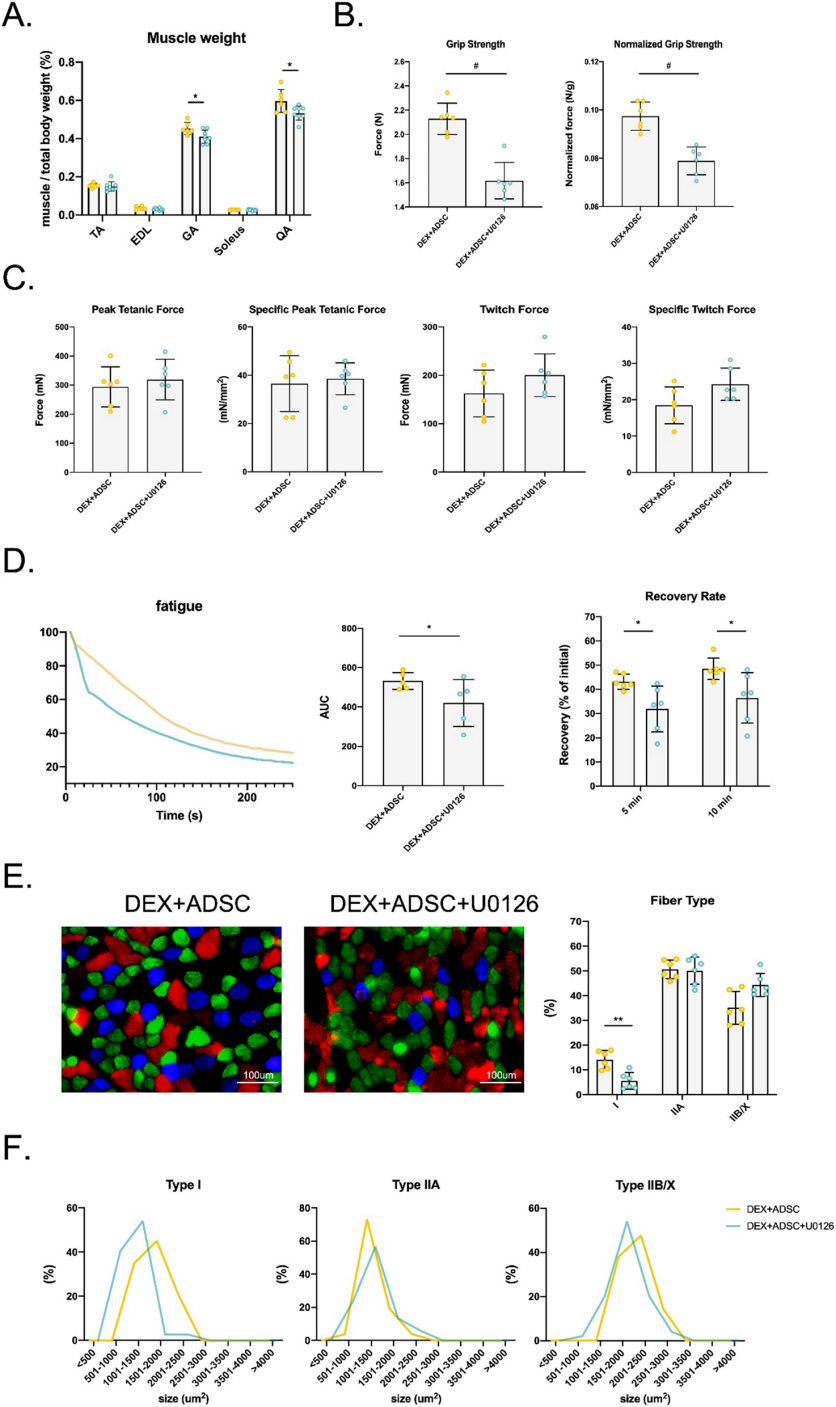
The effect of ERK1/2 inhibition on muscle quality in ADSCs-treated muscle atrophy mice. **A** Quantification of muscle wet weight of TA, EDL, GA, Soleus and QA muscle wet weight. **B** Hindlimb grip strength and normalised by whole body weight grip strength. **C** Ex vivo GA muscle tetanic and twitch fore with or without normalised by GA muscle CSA. **D** GA muscle fatigability and recovery rate measured by the contraction force of GA muscle after 5 min and 10 min of fatigue. **E** Immunofluorescence staining and quantification of fibre types in GA muscle. **F** Distribution of CSA of GA fibre. *n* = 6 per group. Statistical analysis are performed using t-test, with significance set at *P* < 0.05 (*P < 0.05, **P < 0.01, ***P < 0.005, #*P* < 0.001)

To illustrate the role of ERK1/2 signalling in ADSC treatment, U0126 was used to inhibit the activation of ERK1/2 in Dex-induced mice. First, we confirmed that ADSCs were not be able to activate ERK1/2 expression after U0126 injection. Muscle wet weight of GA and QA were partially decreased (Fig. 6A). Forelimb grip strength was reduced after ERK1/2 inhibition (Fig. 6B). However, ex vivo muscle functional test showed that ERK1/2 inhibition barely affected the contraction of GA muscle (Fig. 6C). After continued stimulation, GA muscle from ERK1/2 inhibitor mice became fatigued faster and had slower recovery (Fig. 6D). As described above, fatigue resistant ability is highly controlled by oxidative type I muscle fibre. As expected, the number of type I muscle fibre decreased after ERK1/2 inhibition (Fig. 6E). Distribution of fibre CSA showed that U0126 shifted type I muscle fibre into a smaller size but not type II muscle fibres (Fig. 6F). In conclusion, U0126 can partially inhibit the effects of ADSC especially on improving the anti-fatigue ability and increasing type I muscle fibres.

## Discussion

In this study, we reported that ADSCs could alleviate muscle wasting induced by Dex, especially the function of skeletal muscles. Hindlimb and forelimb muscle functions were enhanced by ADSC treatment, as demonstrated by the grip strength test and ex vivo muscle functional test. In the ex vivo muscle functional test, ADSC treatment improved the twitch force and tetanic force, which indicated better contraction response to force stimulation and showed better anti-fatigue ability. This correlation between anti-fatigue and muscle fibre types correspond with the results shown in previous study [35]. As Type I fibres show more fatigue-resistance compared with fast-twitch fibres, the increase in size and number of type I fibres in GA muscle after ADSC treatment in this study explained the improvement of anti-fatigue ability and recovery rate.

In addition to better anti-fatigue ability, transplantation of human MSC has been found to induce muscle regeneration in animals with muscle atrophy and reduce the expressions of muscle atrophy-associated genes such as Atrogin-1 and Murf-1 [36]. Likewise, our results suggested that muscle mass and functions were significantly improved after ADSC transplantation in mice with Dex-induced muscle atrophy. Besides, findings of in vivo and in vitro experiments suggested that the expression of Atrogin-1 and Murf-1 was inhibited upon ADSC treatment, indicating that ADSC administration can rescue muscle atrophy by regulating the expression of muscle atrophy-associated genes. Both Atrogin-1 and Murf-1 are known to be regulated by the phosphoinositide-3-kinase (PI3K)/Akt signalling pathway [37], which is one of the main signalling pathways involved in Dex-induced muscle atrophy [33, 34]. PI3K/Akt pathway, activated by insulin and IGF1, regulates protein synthesis and degradation, cellular proliferation and survival. Akt inhibits the FoxO3a transcription factor, which is responsible for protein degradation. FoxO3a contributes to the loss of skeletal muscle protein regulated in lysosomal and proteasomal pathways [38–40]. In vivo studies have shown that the phosphorylated level of PI3K/Akt pathway was significantly decreased in Dex-induced muscle atrophy, whereas FoxO3a expression was increased, and E3 ubiquitin ligases were activated [41, 42].

In the present study, gene expression analysis of ADSC treatment in Dex-induced muscle atrophy mouse model identified significant increase in activity of MAPKs, including JNK, ERK1/2 and p38. While the effect of JNK and p38 on various skeletal muscle-associated processes, including proliferation, differentiation and response to contraction and stretch were well documented [43], the effect of ERK1/2 adds a new perspective for understanding the underlying mechanism of its beneficial effect on skeletal muscle qualities. There are few studies suggesting the function of ERK1/2 in regulating skeletal muscle mass and function [44, 45]. However, the correlation between ADSCs and ERK1/2 still unknown. In this study, we linked the function of ADSCs, ERK1/2 signalling pathway and muscle qualities together. Our findings correlate to recent study showing similar results that ERK1/2 signalling pathway has a positive effect on muscle mass and functions in muscle atrophy animal and cell models [44]. We also found that the activation of ERK1/2 signalling pathway by ADSCs is associated with higher number of type I muscle fibres, which improved the anti-fatigue ability of GA muscle, which is consistent with previous study showing that ERK1/2 regulate fast to slow muscle fibre type switching [45]. This is the first study showing the ability of ADSCs in regulating ERK1/2 signalling pathway and further controlling the muscle fibre type switching.

Cell-free-based therapy has drawn interest for its advantages in overcoming the drawback of cell-based therapy [46]. Recent study on cell-free-based therapy has shown that skeletal muscle regeneration is promoted ADSC-derived secretome [47], which is a cocktail of multiple factors and extracellular vesicles, including small exosomes and large microvesicles [48]. Hence, we investigated the effect of ADSC-derived exosome on Dex-induced in vitro sarcopenia model. However, no significant beneficial effects were found. Moreover, similar results as ADSCs were found after inhibition of ADSC to release exosome. Thus, ADSCs might exert their functions in Dex-induced muscle atrophy model through other secretome rather than exosome. According to previous studies, stromal-cell-derived factor 4 (SDF4) found in human ADSC secretome could activate ERK1/2 signalling pathway through the interaction with CXCR4 [49–51]. Besides, studies suggested that MSC secretomes, SDF4, FGF19, could also activate ERK1/2 signalling pathway and are key factors for regulating skeletal muscle mass and functions [52]. These findings support the development of cell-free-based therapy. However, further studies are required to fully understand the mechanism of ADSC secretome on muscle recovery and regeneration. For future translational research, it is important to note that Dex is often used for patients with inflammatory conditions and other clinical problems, which may present confounding effect of pre-existing medical conditions.

## Conclusion

This study provided evidence that ERK1/2 signalling pathway participated in systemic administration of ADSCs for Dex-induced muscle atrophied mice, which alleviate muscle wasting including with increased number of type I fibre, stronger muscle strength, faster recovery rate and more anti-fatigue ability, which can further support the development of pharmaceutical intervention for muscle atrophy. However, there are other mechanisms that may contribute to these effects which cannot be excluded. Therefore, further studies are required to confirm the specificity and elucidate the exact mechanism of action of this pathway, in order to provide more conclusive evidence.

### Supplementary Information

**Additional file 1.** **Figure S1.** Original blot of ERK1/2 protein expression for in vitro *muscle* and in vivo cell samples.

## Data Availability

The RNA-sequencing data sets generated and/or analysed during the current study are available in The Sequence Read Archive (SRA) NCBI, PRJNA953181 (https://www.ncbi.nlm.nih.gov/sra/PRJNA953181).

## Abbreviations

ADSCs: Adipose-derived mesenchymal stem cells
Atrogin-1/MAFbx: Muscle atrophy F-box
CSA: Cross-sectional area
DEGs: Differentially expressed genes
Dex: Dexamethasone
DXA: Dual-energy X-ray absorptiometry
EDL: Extensor digitorum longus
e-IF4G-1: Eukaryotic translation initiation factor 4 gamma 1
Foxo3a: Forkhead box O3
GA: Gastrocnemius
H&E: Hematoxylin and eosin
KEGG: Kyoto encyclopedia of genes and genomes
MAPK: Mitogen-activated protein kinases
MSCs: Mesenchymal stem cells
Mstn: Myostatin
MuRF-1: Muscle RING finger 1
p70s6k: Ribosomal protein S6 kinase beta-1
QA: Quadriceps femoris
TA: Tibialis anterior
