# Supplementary material for: Mesenchymal stem cells alleviate dexamethasone-induced muscle atrophy in mice and the involvement of ERK1/2 signalling pathway
Source: Stem Cell Res Ther. 2023 Aug 4;14:195. doi: 10.1186/s13287-023-03418-0 (PMC10403871; doi:10.1186/s13287-023-03418-0)
Supplement: Supplementary file 1 — Additional file 1. Figure S1. Original blot of ERK1/2 protein expression for in vitro muscle and in vivo cell samples. [file 13287_2023_3418_MOESM1_ESM.pptx]

## Slide 1
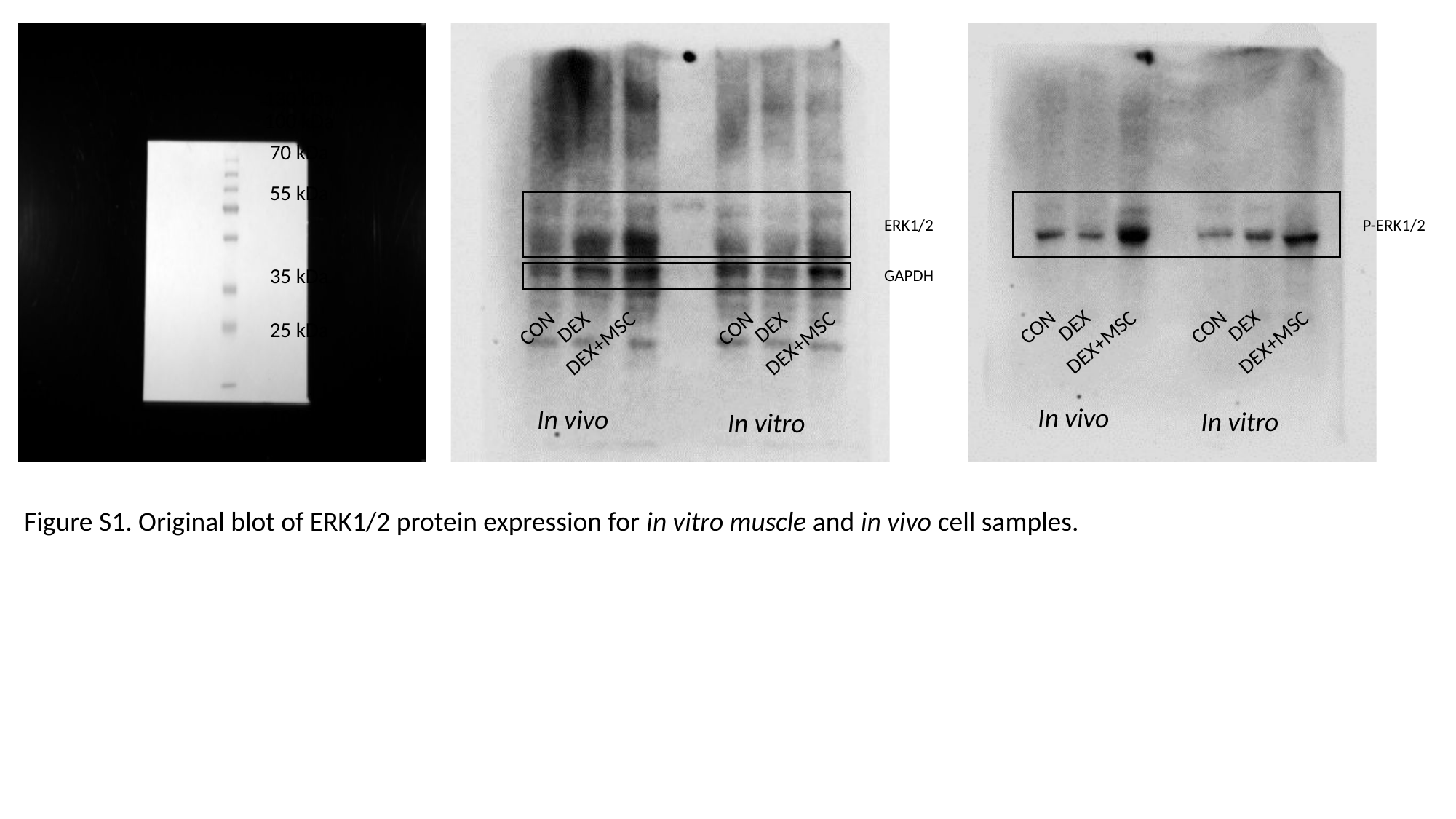

250 kDa
130 kDa
100 kDa
70 kDa
55 kDa
35 kDa
25 kDa
10 kDa
ERK1/2
GAPDH
DEX
DEX
CON
CON
DEX+MSC
DEX+MSC
In vivo
In vitro
P-ERK1/2
DEX
DEX
CON
CON
DEX+MSC
DEX+MSC
In vivo
In vitro
Figure S1. Original blot of ERK1/2 protein expression for in vitro muscle and in vivo cell samples.
